# Facility-based delivery in the context of Zimbabwe’s HIV epidemic – missed opportunities for improving engagement with care: a community-based serosurvey

**DOI:** 10.1186/s12884-015-0782-y

**Published:** 2015-12-17

**Authors:** Raluca Buzdugan, Sandra I. McCoy, Karen Webb, Angela Mushavi, Agnes Mahomva, Nancy S. Padian, Frances M. Cowan

**Affiliations:** University of California Berkeley, School of Public Health, 779 University Hall, MS 7360, Berkeley, CA 94720 USA; University College London, London, United Kingdom; Organisation for Public Health Interventions and Development Trust, 20 Cork Road, Belgravia, Harare Zimbabwe; Ministry of Health and Child Care, Harare, Zimbabwe; Elizabeth Glaser Pediatric AIDS Foundation, 107 King George Road, Avondale, Harare Zimbabwe; Centre for Sexual Health and HIV/AIDS Research Zimbabwe, 9 Monmouth Road, Avondale West, Harare Zimbabwe

**Keywords:** Facility-based delivery, Home-based delivery, Maternal and child health, Prevention of mother-to-child transmission of HIV, HIV, Zimbabwe

## Abstract

**Background:**

In developing countries, facility-based delivery is recommended for maternal and neonatal health, and for prevention of mother-to-child HIV transmission (PMTCT). However, little is known about whether or not learning one’s HIV status affects one’s decision to deliver in a health facility. We examined this association in Zimbabwe.

**Methods:**

We analyzed data from a 2012 cross-sectional community-based serosurvey conducted to evaluate Zimbabwe’s accelerated national PMTCT program. Eligible women (≥16 years old and mothers of infants born 9–18 months before the survey) were randomly sampled from the catchment areas of 157 health facilities in five of ten provinces. Participants were interviewed about where they delivered and provided blood samples for HIV testing.

**Results:**

Overall 8796 (77 %) mothers reported facility-based delivery; uptake varied by community (30–100 %). The likelihood of facility-based delivery was not associated with maternal HIV status. Women who self-reported being HIV-positive before delivery were as likely to deliver in a health facility as women who were HIV-negative, irrespective of when they learned their status - before (adjusted prevalence ratio (PR_a_) = 1.04, 95 % confidence interval (CI) = 1.00–1.09) or during pregnancy (PR_a_ = 1.05, 95 % CI = 1.01–1.09). Mothers who had not accessed antenatal care or tested for HIV were most likely to deliver outside a health facility (69 %). Overall, however 77 % of home deliveries occurred among women who had accessed antenatal care and were HIV-tested.

**Conclusions:**

Uptake of facility-based delivery was similar among HIV-infected and HIV-uninfected mothers, which was somewhat unexpected given the substantial technical and financial investment aimed at retaining HIV-positive women in care in Zimbabwe.

## Background

Although decreasing in many regions of the world, maternal and infant mortality rates are still far from the Millennium Development Goal targets for 2015. [1, 2] In 2013 almost 300,000 maternal deaths [1] and over 2 million early neonatal deaths occurred globally, [2] the majority in developing countries. With up to 40 % of maternal and newborn deaths and stillbirths occurring within 24 hours of birth, [3] skilled birth attendance has been strongly promoted. [4] Although there have been some successful initiatives to train existing traditional birth attendants in the community, [5, 6] in developing countries skilled birth attendance is largely synonymous with facility-based delivery. [7]

In addition to its role in maternal and neonatal health, facility-based delivery is recommended for the prevention of mother-to-child HIV transmission (PMTCT). Specifically, effective PMTCT requires the uptake and retention of pregnant women in a cascade of services including HIV testing, early uptake and adherence to antiretroviral (ARV) prophylaxis, safe obstetric practices and infant feeding counseling. [8] Vertical transmission of HIV during pregnancy, labor and breastfeeding can be reduced from 15 to 45 % to <1 % with appropriate and timely ARV-based interventions. [9] In the absence of ARVs, the greatest transmission risk occurs in the intrapartum period. [9] Home delivery has been cited as a contributing factor to limiting coverage and retention of mothers in PMTCT programs in sub-Saharan Africa. [10, 11] For example, a recent review found that home delivery is a key factor contributing to poor adherence to short-course ARV prophylaxis for PMTCT in sub-Saharan Africa. [12, 13] Facility-based delivery remains critical under Option B+ (the current WHO-recommended PMTCT guidelines whereby all HIV-infected pregnant women receive lifelong ART), [8] given the high loss to follow-up after initiating ART. [14]

Zimbabwe, where our study is based, has high maternal and infant mortality; the 2012 census reports 525 maternal deaths per 100,000 live births and 64 infant deaths per 1000 births. [15] Moreover, 12 % of pregnant women are HIV-positive [16] and at least 9 % of their infants become HIV-infected. [17] Only 65 % of births in Zimbabwe take place in health facilities according to the 2010–11 Zimbabwe Demographic Health Survey (ZDHS). [16] The 2010–11 ZDHS data also report that <5 % of women who deliver outside health facilities do so in the presence of a skilled attendant. [16] In the 1980s and 1990s traditional birth attendants were officially recognized and trained as skilled midwives; [18] however, since then their training has been actively discouraged. [19] Women from rural areas, who do not access antenatal care (ANC), who have high parity, with low levels of education, or who are poor are more likely to deliver at home. [16]

Due to the documented increased risk of non-adherence to ARVs and mother-to-child transmission associated with home-based delivery, [10–13] delivering in a health facility is particularly critical for HIV-infected women and health staff are instructed to emphasize its importance during ANC. Hence, women who know they are HIV-infected should be more likely than their counterparts to deliver in a health facility because of the enhanced counseling they receive on the importance of facility-based delivery for safe delivery and PMTCT. However, little is known about whether learning one’s HIV status affects one’s decision to deliver in a health facility or not, and the limited existing evidence is mixed. [20, 21] This evidence gap is particularly relevant in the context of substantial technical and financial investments in national PMTCT programs in developing countries in recent years, including in Zimbabwe. Moreover, a better understanding is needed of the key points of contact with the healthcare system for women who do *not* deliver in health facilities, as these represent missed opportunities to inform women of the importance and benefits of facility-based delivery. Using representative survey data from Zimbabwe, [17, 22] here we examine: 1) the uptake of facility-based delivery and community-level variations in uptake in Zimbabwe in 2010–2011, 2) the association between HIV status and uptake of facility-based delivery, and 3) the prevalence of women who did not deliver in health facilities, depending on whether they accessed ANC and were tested for HIV.

## Methods

We analyzed data from a 2012 baseline cross-sectional survey conducted to evaluate Zimbabwe’s accelerated national PMTCT program implemented in 2011. The objective of that evaluation was to assess the population-level impact of the PMTCT program on MTCT and HIV-free child survival at 9–18 months postpartum. [22] The methods have been published in detail elsewhere. [17, 23, 24]

In brief, infants (alive or deceased) born 9–18 months prior (henceforth ‘index babies’) and their mothers or caregivers (≥16 years old) were eligible for the community-based baseline survey. The infants’ age range was chosen to meet the objectives of the impact evaluation. [17] For this analysis, we excluded data regarding caregivers (*n* = 349, 3.9 % of 9018 participants) and only analyzed data on living biological mothers who were present at the time of the survey (*n* = 8662), as data collected about deceased (*n* = 55) and unavailable mothers (*n* = 294) did not include information about their place of delivery.

The survey was conducted in April-September 2012 in five of Zimbabwe’s ten provinces (Harare, Mashonaland West, Mashonaland Central, Manicaland, Matabeleland South). These regions include both major ethnic groups (i.e., Shona, Ndebele), some of the largest cities in Zimbabwe, and rural areas with higher and lower HIV prevalence. Study participants were identified using a two-stage stratified cluster design. Firstly, of the 699 health facilities offering PMTCT services in these five provinces, we randomly selected 157 facilities, proportionate to the number of facilities in each district. Secondly, we identified all eligible infants from the catchment areas of these 157 facilities and sampled a known fraction of them proportionate to the size of the target population in each catchment area. Eligible mother-infant pairs were identified based on information pooled from community health workers and immunization registers from selected and neighboring facilities (to identify women residing in sampled facilities who accessed services at facilities nearby). Further, those mothers identified using community health workers and immunization registers were asked to identify other infants in their neighborhood who were born in the previous two years.

### Data collection

Trained interviewers visited the houses of potentially eligible mother-infant pairs (identified as explained above), verified their eligibility, administered the questionnaire in the participant’s preferred language (English, Shona or Ndebele) and collected dried blood spot samples for HIV antibody testing for infants and mothers. Specifically, participating mothers answered anonymous interviewer-administered questionnaires, capturing the mothers’ demographic characteristics, healthcare utilization and place of delivery for the index baby. Maternal samples were stored at room temperature and tested for HIV-1 antibody in batches, using AniLabsytems EIA kit (AniLabsystems Ltd, OyToilette 3, FIN-01720, Vantaa, Finland). Positive specimens were confirmed using Enzygnost Anti-HIV 1/2 Plus ELISA (Dade Behring, Marburg, Germany) and discrepant results were resolved by Western Blot. [25]

### Key variables

#### Facility-based delivery

To assess the place of delivery of the index baby, participating mothers were asked “where did you give birth to your baby?”. We categorized women into two groups: i) mothers who delivered in healthcare facilities e.g., clinic, health center, hospital (henceforth ‘facility-based delivery’) and ii) mothers who delivered at home or elsewhere e.g., someone else’s home (henceforth ‘home-based delivery’).

#### Maternal HIV status

For this paper, we measured maternal HIV status in two ways. Firstly, our analyses used the mothers’ *self-reported HIV status* before delivery, based on the assumption that women’s healthcare behavior could only have been influenced by their known HIV status at the time of delivery (rather than unknown and laboratory-assessed status). Self-reported HIV status distinguished between mothers who did not know their status before delivery, those who reported they were HIV-negative before delivery, those who reported they were HIV-infected before the pregnancy, and those who learned they were HIV-infected while pregnant with the index baby. Secondly, we examined the association between the uptake of facility-based delivery and the mother’s laboratory-assessed HIV status at the time of the survey.

#### Healthcare utilization

We examined utilization of health services during the pregnancy (i.e., ANC, HIV testing) as these are key services preceding labor and delivery and thus represent possible opportunities to inform women of the benefits of facility-based delivery.

#### Covariates

We examined several covariates for inclusion in the multivariate models as potential confounders: province of residence, urban/rural status, age, highest educational level, religion, marital status, parity, the decision-maker regarding the place of delivery (i.e., mother, father, other), the sex of the person who makes important household decisions (i.e., female, male, both), the number of sellable assets present in the household (i.e., livestock, bicycle, motorcycle, car/truck, scotch cart, wheel barrow, phone, radio, television) and household-level food security as another indicator for household economic status. Household food security was assessed based on questions from the Household Food Insecurity Access Scale on anxiety and uncertainty about household food supply, insufficient food quality and insufficient food intake; we distinguished between three categories: food security, moderate food insecurity and severe food insecurity. These variables have been selected as covariates for inclusion in the multivariate models because previous studies have shown these factors to be associated with uptake of facility-based delivery in sub-Saharan Africa. [26]

### Data analysis

First, we described the uptake of facility-based delivery in Zimbabwe at the individual and community levels. At the individual level, we estimated the uptake of facility-based delivery in our sample and by province. We also assessed the aggregate-level uptake of facility-based delivery within each catchment area, the proxy for community. Community-level analyses include data from 156 catchment areas; we excluded one catchment area where only 4 mother-infant pairs were recruited in the study.

Second, we examined the association between maternal HIV status and the uptake of facility-based delivery through univariate, bivariate and multivariate analyses. We constructed unadjusted and adjusted Poisson regression models with uptake of facility-based delivery (i.e., yes, no) as the outcome and self-reported HIV status before delivery as the exposure. In Poisson models with cross-sectional data, the exponentiated parameter estimates represent prevalence ratios, a conservative and more interpretable measure of association (compared to odds ratio) if the outcome is common, [27–30] as is the case for facility-based delivery (77 %). In building the adjusted model we checked for statistical interactions between each covariate and the uptake of facility-based delivery, as well as multicollinearity between the variables included in the models.

Third, we explored healthcare utilization in our sample of recent mothers. Specifically, we distinguished between six categories of women, corresponding to the six possible combinations of the following two variables: receipt of ANC during the pregnancy (i.e., yes, no) and self-reported HIV status before delivery (i.e., not tested, HIV-negative, HIV-positive). For each of these six groups we computed the absolute and relative frequency of home-based deliveries. All analyses were conducted in STATA 12; we used the STATA *svy* commands, which allowed us to weight the data to account for the two-stage stratified cluster design and the survey non-response, and to adjust for catchment area-level clustering.

### Human subjects protection

The Medical Research Council of Zimbabwe and the ethics committees of the University of California, Berkeley and University College London approved the study protocol. Written informed consent was obtained from all participants prior to their participation. All participants received a gift (i.e., laundry soap and petroleum jelly) worth approximately $5USD. Women were able to receive their HIV test results at the local health facility up to 3 months after the survey, using a card with their unique identifier barcoded.

## Results

We visited 42,995 households; 21,047 households (49 %) had at least one eligible mother-infant pair. In 571 households (1.3 %) we were not able to assess whether an eligible mother-infant pair lived there or not. Overall, we identified 21,205 eligible mother-infant pairs of which 9184 (43 %) were invited to participate in the survey. Of these, 9080 (98.9 %) mother-infant pairs consented to the questionnaire. After excluding corrupted files, questionnaires were available regarding 9018 (98.2 %) mother-infant pairs. We limited the analysis for this paper to 8662 living biological mothers who were present at the time of the survey.

### Uptake of facility-based delivery

Overall, 76.8 % of all mothers delivered in a health facility, ranging between 69 % and 92 % by province (Table 1). There is variation in uptake of facility-based delivery between catchment areas (range: 30 to 100 % of mothers within a catchment area delivered in a health facility; mean 74.4; data not shown).

**Table 1:** Frequency of facility-based deliveries by province, estimated by the 2012 survey and the 2010–2011 ZDHS

	2012 PMTCT survey^a^ (deliveries between 10/2010 and 11/2011)		2010-2011 ZDHS^b^ (deliveries between 09/2005 and 03/2011)	
	*N*	% (95 % CI)	*N*	%
Harare	1535	92.4 (89.1–94.8)	826	82.7
Manicaland	3564	75.3 (71.4–78.8)	843	60.9
Mashonaland Central	1504	70.5 (65.8–74.8)	603	50.3
Mashonaland West	1339	69.0 (63.2–74.2)	701	52.6
Matabeleland South	855	78.2 (72.4–83.0)	273	69.3
Mashonaland East	Not surveyed		530	59
Matebeleland North	Not surveyed		265	63.5
Midlands	Not surveyed		701	63.4
Masvingo	Not surveyed		627	73.4
Bulawayo	Not surveyed		227	88.3
Total (5 provinces selected in our survey)	8796	76.8 (73.9–79.5)	3246	63.4
Total (all 10 Zimbabwean provinces)	Not estimated		5596	65.1

*PMTCT* prevention of mother to child transmission of HIV, *ZDHS* Zimbabwe Demographic Health Survey, *CI* confidence interval

^a^ Percentage of facility-based deliveries among all live births in the 9–18 months prior to the survey (weighted estimates)

^b^ Percentage of facility-based deliveries among all live births in the five years prior to the survey

### Association between maternal HIV status and uptake of facility-based delivery

Uptake of health facility delivery was similar among HIV-infected and HIV-uninfected mothers (Table 2). Moreover, women who self-reported being HIV-positive were just as likely to deliver in a health facility as women who were HIV-negative, irrespective of whether they reported that they found out their status before the pregnancy (adjusted prevalence ratio (PR_a_) = 1.04, 95 % confidence interval (CI) = 1.00–1.09) or during the pregnancy with the index baby (PR_a_ = 1.05, 95 % CI = 1.01–1.09). However, only 44 % of mothers with unknown self-reported HIV status (representing 7.8 % of all mothers) delivered in a health facility (PR_a_ = 0.60, 95 % CI = 0.54–0.68 compared to women who self-reported being HIV-negative). Results were similar when examining the association between uptake of facility-based delivery and women’s laboratory-confirmed HIV status at 9–18 months postpartum; HIV-infected women (representing 12 % of all mothers) were just as likely as HIV-uninfected mothers (representing 86 % of all mothers) to deliver in a health facility (76 vs. 77 %, PR_a_ = 1.02, 95 % CI = 0.98–1.07, p = 0.234; data not shown).

**Table 2:** The association between maternal HIV status and uptake of facility-based delivery, Zimbabwe 2012

	Overall frequency		Uptake of facility-based delivery				
	*N*	%	%	PR_u_	95 % CI	PR_a_	95 % CI^a^
Self-reported HIV status				(*p* < 0.001)		(*p* < 0.001)	
Not tested / unknown status before delivery	686	(7.8)	44.4	0.56	(0.49–0.64)	0.60	(0.54–0.68)
Self-reported HIV-negative before delivery	7067	(80.3)	79.3	1.00		1.00	
Self-reportedHIV-positive before pregnancy	584	(6.6)	82.2	1.04	(0.99–1.09)	1.04	(1.00–1.09)
Self-reported HIV-positive during pregnancy	459	(5.2)	80.4	1.01	(0.97–1.06)	1.05	(1.01–1.09)

*PR*_*u*_ unadjusted prevalence ratio, *PR*_*a*_ adjusted prevalence ratio, *CI* confidence interval

^a^Regression model of 8685 biological women, adjusted for the clustered study design. In addition to the variable listed in the table, the model includes province, urban vs. rural status, maternal age, education, religion, number of household assets, household-level food security status, lifetime births, marital status, sex of household head, decision maker of place of delivery

### Healthcare utilization of women who delivered outside a health facility

**Fig. 1:**
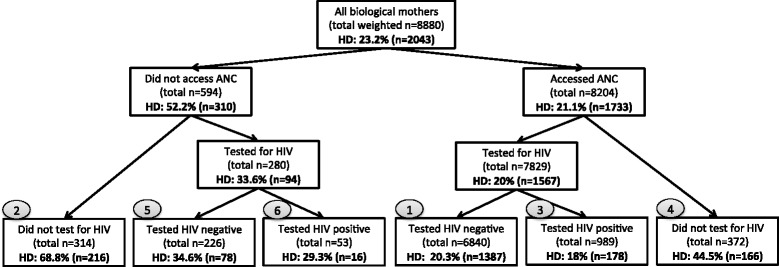
Prevalence and frequency of home-based delivery stratified by whether mothers accessed ANC and were HIV-tested. HD = home-based delivery (i.e., delivery at mother’s own home, someone else’s home, any other place outside a health facility), ANC = antenatal care. HIV status is measured by self-reported test result before delivery. All the variables used to generate this Figure are self-reported. *Note 1:* The grey circles indicate the rank of each category, in terms of its absolute contribution to the total number of home-based deliveries. For example, although only 20 % of women who accessed antenatal care and tested HIV-negative delivered outside a health facility, these women represent 68 % of the total sample of home-based deliveries (*n* = 1387 of 2043). *Note 2*: The figure presents weighted n values, which have been rounded to the nearest whole number

We examined the absolute and relative frequency of mothers who delivered at home, depending on whether they accessed ANC and were HIV-tested. Specifically, on the bottom row of Fig. 1 are listed six categories of women, corresponding to the six possible combinations of receipt of ANC during the pregnancy (yes/ no) and self-reported HIV status before delivery (not tested/ HIV-negative/ HIV-positive). For each of these categories, we provide the frequency of home-based delivery (in the boxes) and its rank in terms of its absolute contribution to the total number of home-based deliveries (in the grey circles).

Women who accessed ANC and tested HIV-negative before delivery represent 68 % of all mothers with home-based deliveries (*n* = 1387 of 2043); this category of women ranks first in terms of its absolute contribution to the total number of home-based deliveries. However, the proportion of women who delivered at home ranged between 18 % of women who attended ANC and tested HIV-positive to 69 % of women who had not accessed ANC or tested for HIV. Proportionately, women were most likely to deliver at home if they did not receive any or partial prenatal services (i.e., 69 % of women who had not accessed ANC or tested for HIV, 45 % of women who accessed ANC but did not test for HIV). Proportionately, women were least likely to deliver at home if they both attended ANC and received the results of an HIV test, regardless of whether it was HIV-positive (18 %) or HIV-negative (20 %).

## Discussion

In this paper we examined the uptake of facility-based delivery among women recruited in a large survey of mother-infant pairs conducted in Zimbabwe in 2012, one of the first studies to assess population-level estimates of HIV-free infant survival accounting for breastfeeding-related transmission using a community-based sample. [17, 22] We found that 77 % of mothers reported facility-based delivery, which varied considerably by community. The likelihood of facility-based delivery was not associated with self-reported maternal HIV status before delivery. Mothers who neither accessed ANC nor tested for HIV were the most likely to deliver at home (69 %) compared to other patterns of service utilization, however most home-based deliveries were among women who both accessed ANC and were tested for HIV infection. Although only 20 % of mothers who accessed ANC and reported testing HIV-negative delivered at home, these represented over two thirds of all home-based deliveries.

Our estimate of the overall uptake of facility-based delivery (77 %) is higher than the 2010–2011 ZDHS estimate (65 %). [16] Differences in sampling strategies between the two studies notwithstanding, this is likely indicative of the difference in survey timing and target populations i.e., our estimate included live births during the 9–18 months (2010–2011) prior to the survey in five provinces, while the ZDHS estimate included all live births to women in all ten Zimbabwean provinces in the five years (2005–2011) preceding the survey. Although our survey covered only half of the nation’s provinces, our estimate is likely a more accurate indicator of more *recent* uptake of facility-based delivery in Zimbabwe. The difference between the two estimates may suggest that the declining trend of deliveries in health facilities documented by ZDHS data (72 % in 1999, 68 % in 2005–2006, 65 % in 2010–2011) [16] has ended or reversed and uptake of facility-based delivery is now increasing.

Uptake of facility-based delivery was similar among HIV-infected and HIV-uninfected mothers, as also documented in Kenya. [31] However, in Rwanda 2002–2203 program data indicated that facility-based delivery in PMTCT programs is 2.7 times higher than in general population. [21] Given Zimbabwe’s substantial investment to retain HIV-infected women in care in recent years (e.g., approximately $USD 40 million in 2012), [32] and the counseling messages that strongly advise women who test HIV-positive during ANC to deliver in a facility, one might expect HIV-infected women (at least those who know their HIV status) to deliver in health facilities in larger numbers than HIV-negative women. At the same time, because they are expected to deliver in a facility, HIV-infected women who have not disclosed their status to community members may be reluctant to deliver in facilities to avoid disclosure, stigma and discrimination which continue to be important deterrents to uptake of HIV services Zimbabwe. [33] Non-disclosure of HIV status has been linked to low facility-based delivery, [31] poor uptake of PMTCT [31, 34–36] and maternal and child health services [37] in developing country settings. Further, HIV-related stigma was associated with low uptake of facility-based delivery in Kenya. [20]

We find that women who reported they did not know their HIV status before delivery were significantly less likely to deliver in a health facility than those who reported knowing their status (44 vs. 80 %). The laboratory-confirmed HIV prevalence among mothers with unknown self-reported HIV status is 13.6 %, similar to the prevalence among all mothers in our sample (13 %). Assuming that women’s perceived risk of HIV infection is consistent to their actual HIV status, this suggests that the low uptake of facility-based delivery among women with unknown self-reported HIV status may not be an indication of perceived risk of testing HIV-positive, although available data do not allow us to further examine this hypothesis. Although women with unknown self-reported HIV status are the least likely to deliver in health facilities, particularly if they did not access ANC, they represent only 19 % of home-based deliveries overall.

The large majority of women who delivered at home accessed ANC: of the 2043 women who delivered at home included in the survey, 1387 accessed ANC and tested HIV-negative compared to only 216 who did not access ANC and did not test for HIV, suggesting that it should be possible to reach *most* women who are at risk of home-based delivery through their contact with health services. Hence, community and clinic-based staff need to continue to convey information about the importance and benefits of facility-based delivery for *all* women during pregnancy. Additionally, other approaches could further convince women to deliver in a health facility. Financial incentive schemes, [38, 39] community referral/ transport systems, [39] use of mobile technology, [39] birth preparedness and planning, [40] and male involvement are promising interventions for improving uptake of facility-based delivery, although further evaluations of their effect on maternal and infant mortality are needed.

In over half (57 %) of the communities, fewer than 80 % of recent mothers had delivered in a health facility. In addition, we found considerable variation in the community-level uptake of facility-based delivery, suggesting that there may be important community-specific factors in favor or against the practice of delivery in health facilities (e.g., social norms, religious norms), and/or facility-level factors including perceived quality of care among community members. Our survey collected limited information on community and facility characteristics, hence we cannot explore this hypothesis in detail. Future studies may want to explore community-level factors in more detail in order to better understand how these affect beliefs and practices related to childbearing.

The primary intention of this survey was not to assess mothers’ place of delivery. Consequently, various factors related to facility-based delivery were not examined in this study, and the place of delivery, healthcare utilization variables, as well as the self-reported HIV status before delivery were not verified against medical records. Moreover, these self-reported data relate to behaviors that participating mothers engaged in 9–18 months prior to the survey; hence, these data are subject to recall and social desirability bias. Nonetheless, the key variables included in our analyses relate to major events in the participants’ lives (e.g., birth of their child, finding out their HIV status), which are inherently memorable. Further, data were collected anonymously, which should minimize social desirability bias. Notably, the analysis excluded women who had died by the time of the survey, who may have been more likely to be HIV-infected. Although large-scale and representative, the survey was conducted in five of Zimbabwe’s ten provinces, allowing the possibility that uptake and patterns of facility-based delivery are substantially different in the non-surveyed provinces. Lastly, while we identified eligible mother-infant pairs using multiple approaches, isolated marginalized women may be underrepresented.

Further research is required to understand the ‘tipping point’ barriers and facilitators for service uptake faced by both HIV-negative and HIV-positive women to inform design of innovative interventions to increase demand and uptake of maternal health services and improve retention across the PMTCT continuum in the drive to virtual elimination of pediatric HIV. To meet its national goal of 85 % uptake of facility-based delivery by 2019, Zimbabwe aims to establish mothers’ waiting homes (residential facilities where pregnant women at risk of complications or who face distance as a barrier to uptake can await onset of labor) in all district hospitals by 2015. [41] Other interventions are underway through the Ministry of Health and Child Care and its partners to increase access and uptake to quality health services for women and children. Implementation science research is required to assess the impact of such efforts on uptake of facility-based delivery and on health outcomes of women and children, including PMTCT-related outcomes.

## Conclusion

Our study found that 77 % of recent mothers in Zimbabwe delivered in a health facility. There was similar uptake of facility-based delivery regardless of maternal HIV status. The majority of women who delivered at home had previously accessed services suggesting missed opportunities for encouraging uptake of facility-based delivery. Women who failed to access ANC were the least likely to have tested for HIV and to deliver in a facility, suggesting that community health services need to intensify efforts to link these women to care.

## Abbreviations

ANC: antenatal care
ARV: antiretroviral
PMTCT: prevention of mother-to-child HIV transmission
ZDHS: Zimbabwe Demographic Health Survey

## References

[1] Kassebaum NJ, Bertozzi-Villa A, Coggeshall MS, Shackelford K a, Steiner C, Heuton KR (2014). Global, regional, and national levels and causes of maternal mortality during 1990–2013: a systematic analysis for the Global Burden of Disease Study 2013. Lancet.

[2] Wang H, Liddell C a, Coates MM, Mooney MD, Levitz CE, Schumacher AE (2014). Global, regional, and national levels of neonatal, infant, and under-5 mortality during 1990–2013: a systematic analysis for the Global Burden of Disease Study 2013. Lancet.

[3] Lawn JE, Blencowe H, Oza S, You D, Lee ACC, Waiswa P (2014). Every Newborn: progress, priorities, and potential beyond survival. Lancet.

[4] Samarasekera U, Horton R (2014). The world we want for every newborn child. Lancet.

[5] Bang AT, Bang RA, Baitule SB, Reddy MH, Deshmukh MD (1999). Effect of home-based neonatal care and management of sepsis on neonatal mortality: field trial in rural India. Lancet.

[6] Jokhio AH, Winter HR, Cheng KK (2005). An intervention involving traditional birth attendants and perinatal and maternal mortality in Pakistan. N Engl J Med.

[7] Perez F, Aung KD, Ndoro T, Engelsmann B, Dabis F. Participation of traditional birth attendants in prevention of mother-to-child transmission of HIV services in two rural districts in Zimbabwe: a feasibility study. BMC Public Health. 2008;8. 10.1186/1471-2458-8-401 PMC2612666 19061506

[8] World Health Organization (2013). Consolidated guidelines on the use of antiretroviral drugs for treating and preventing HIV infection. Recommendations for a public health approach.

[9] World Health Organization (2010). Antiretroviral drugs for treating pregnant women and preventing HIV infection in infants: recommendations for a public health approach.

[10] Kalembo FW, Zgambo M (2012). Loss to Followup: A Major Challenge to Successful Implementation of Prevention of Mother-to-Child Transmission of HIV-1 Programs in Sub-Saharan Africa. ISRN AIDS.

[11] Sibanda EL, Weller IVD, Hakim JG, Cowan FM (2013). The magnitude of loss to follow-up of HIV-exposed infants along the prevention of mother-to-child HIV transmission continuum of care: a systematic review and meta-analysis. AIDS.

[12] Albrecht S, Semrau K, Kasonde P, Sinkala M, Kankasa C, Vwalika C (2006). Predictors of Nonadherence to Single-Dose Nevirapine Therapy for the Prevention of Mother-to-Child HIV Transmission. J Acquir Immune Defic Syndr.

[13] Kasenga F, Hurtig A-K, Emmelin M (2007). Home deliveries: implications for adherence to nevirapine in a PMTCT programme in rural Malawi. AIDS Care.

[14] Tenthani L, Haas AD, Tweya H, Jahn A, van Oosterhout JJ, Chimbwandira F (2014). Retention in care under universal antiretroviral therapy for HIV-infected pregnant and breastfeeding women (’Option B + ') in Malawi. AIDS.

[15] Zimbabwe National Statistics Agency, Population Census Office. Census 2012 - National Report. Harare, Zimbabwe; 2012. http://www.zimstat.co.zw/sites/default/files/img/publications/Census/CensusResults2012/National_Report.pdf.

[16] Zimbabwe National Statistics Agency, ICF International (2012). Zimbabwe Demographic and Health Survey 2010–11.

[17] Buzdugan R, McCoy SI, Watadzaushe C, Kang Dufour M-S, Petersen M, Dirawo J (2015). Evaluating the impact of Zimbabwe’s prevention of mother-to-child HIV transmission program: population-level estimates of HIV-free infant survival pre-Option A. PLoS One.

[18] Ministry of Health and Child Welfare (1997). Traditional Midwives Training Guidelines.

[19] Choguya NZ (2014). Traditional Birth Attendants and Policy Ambivalence in Zimbabwe. J Anthropol.

[20] Turan JM, Hatcher AH, Medema-Wijnveen J, Onono M, Miller S, Bukusi E a (2012). The role of HIV-related stigma in utilization of skilled childbirth services in rural Kenya: a prospective mixed-methods study. PLoS Med.

[21] Nyankesha E, Mugenzi C, Sibailly T, Munyakaz L. The institutional delivery rate in Prevention of Mother-To-Child Transmission (PMTCT) programs is 2.7 times higher than in general population in Rwanda. XV International AIDS Conference in Bangkok, Thailand. 2004, p. ThPeB7082.

[22] Buzdugan R, McCoy S, Petersen M, Guay L, Mushavi A, Mahomva A, et al. Feasibility of population-based cross-sectional surveys for estimating vertical HIV transmission: data from Zimbabwe. 7th IAS Conference on HIV Pathogenesis, Treatment and Prevention. Kuala Lumpur, Malaysia; 2013.

[23] McCoy SI, Buzdugan R, Ralph LJ, Mushavi A, Mahomva A, Hakobyan A (2014). Unmet Need for Family Planning, Contraceptive Failure, and Unintended Pregnancy Among HIV-infected and HIV-uninfected Women in Zimbabwe. PLoS One.

[24] McCoy SI, Buzdugan R, Padian NS, Engelsman B, Martz TE, Mushavi A (2015). Uptake of services and behaviors in the prevention of mother-to-child HIV transmission (PMTCT) cascade in Zimbabwe. J Acquir Immune Defic Syndr.

[25] US Department of Health and Human Services. Serologic assays for human immunodeficiency virus antibody in dried blood specimens collected on filter paper; 2000.

[26] Moyer C a, Mustafa A (2013). Drivers and deterrents of facility delivery in sub-Saharan Africa: a systematic review. Reprod Health.

[27] Barros AJD, Hirakata VN (2003). Alternatives for logistic regression in cross-sectional studies: an empirical comparison of models that directly estimate the prevalence ratio. BMC Med Res Methodol.

[28] Zoccheto C, Consonni D, Bertazzi PA (1995). Estimation of Prevalence Rate Ratios from Cross-Sectional Data. Int J Epidemiol.

[29] Levin B (1991). Re: “Interpretation and choice of effect measures in epidemiologic analyses”. Am J Epidemiol.

[30] Zocchetti C, Consonni D, Bertazzi P a (1997). Relationship between prevalence rate ratios and odds ratios in cross-sectional studies. Int J Epidemiol.

[31] Spangler S a, Onono M, Bukusi E a, Cohen CR, Turan JM (2014). HIV-Positive Status Disclosure and Use of Essential PMTCT and Maternal Health Services in Rural Kenya. J Acquir Immune Defic Syndr.

[32] Hecht R, Williamson A, Guthrie T, Mbetu T, Ishtiaq A (2014). Zimbabwe Cost and Financing of the Shift to eMTCT Option B+.

[33] O’Brien S, Broom A (2013). Gender, culture and changing attitudes: experiences of HIV in Zimbabwe. Cult Heal Sex.

[34] Gourlay A, Birdthistle I, Mburu G, Iorpenda K, Wringe A (2013). Barriers and facilitating factors to the uptake of antiretroviral drugs for prevention of mother-to-child transmission of HIV in sub-Saharan Africa: a systematic review. J Int AIDS Soc.

[35] Ferguson L (2013). Women’s experiences in services for preventing the mother-to-child transmission of HIV: a literature review [Internet].

[36] Jasseron C, Mandelbrot L, Dollfus C, Trocmé N, Tubiana R, Teglas JP (2013). Non-disclosure of a pregnant woman’s HIV status to her partner is associated with non-optimal prevention of mother-to-child transmission. AIDS Behav.

[37] Turan JM, Nyblade L (2013). Global maternal and child health goals will not be achieved without addressing HIV-related stigma. J Acquir Immune Defic Syndr.

[38] Lim SS, Dandona L, Hoisington JA, James SL, Hogan MC, Gakidou E (2010). India’s Janani Suraksha Yojana, a conditional cash transfer programme to increase births in health facilities: an impact evaluation. Lancet.

[39] Lee AC, Lawn JE, Cousens S, Kumar V, Osrin D, Bhuttae ZA (2009). Linking families and facilities for care at birth: What works to avert intrapartum-related deaths?. Int J Gynaecol Obstet.

[40] Nawal D, Goli S (2013). Birth preparedness and its effect on place of delivery and post-natal check-ups in Nepal. PLoS One.

[41] Government of Zimbabwe (2013). Zimbabwe Agenda for Sustainable Socio-Economic Transformation (Zim Asset); 2013–2018.

